# The use of radiological imaging alongside reverse transcriptase PCR in diagnosing novel coronavirus disease 2019: a narrative review

**DOI:** 10.2217/fmb-2020-0098

**Published:** 2020-07-08

**Authors:** Michael Naguib, Fathi Moustafa, Mustafa Thaer Salman, Nermin Kamal Saeed, Manaf Al-Qahtani

**Affiliations:** ^1^Department of Medicine, Royal college of Surgeons in Ireland - Medical University of Bahrain, Al Sayh Muharraq Governorate, Bahrain; ^2^Department of Medicine, Royal Medical services, Bahrain Defense Force Hospital, Riffa, Bahrain; ^3^Department of Pathology, Head of Microbiology section, Salmaniya medical Complex, Ministry of health, Manama, Bahrain

**Keywords:** chest x-ray, COVID-19, CT scan, diagnostic radiology, virology

## Abstract

The diagnosis of novel coronavirus disease 2019 (COVID-19) has been a challenge in many countries due to nonspecific symptoms and variable incubation period. The current reference test is reverse transcriptase PCR. Many studies have reported high sensitivities of CT scans and suggested that they can be used in the diagnosis of COVID-19 alongside reverse transcriptase PCR. The current data about CT scans are highly variable and incoherent. Therefore, new multicentric studies in different countries are needed to better understand the role of CT scans in COVID-19 diagnosis. In this report, we will discuss the clinical relevance of each test and the current Centers for Disease Control and Prevention and American College of Radiology recommendations regarding the use of imaging in the diagnosis of COVID-19.

SARS coronavirus 2 is the name given to the virus that causes the novel coronavirus disease 2019 (COVID-19). This virus was identified as the causative agent for a number of pneumonia cases reported in Wuhan, China during December 2019 [1]. Initially, COVID-19 was not a remarkably deadly illness, yet it was very contagious [2]. However, mortality rates have been varying and increasing across different countries. For example, the mortality rates reported in China, USA and Italy were 5.5, 6.1 and 14.0%, respectively [3]. Due to its highly contagious nature, the virus has spread very rapidly and countries all around the world have taken aggressive measures in an attempt to limit its spread. On 11 March 2020, the WHO declared COVID-19 as a global pandemic [4]. As of the 7 June 2020, the number of confirmed cases around the world is about 6.8 million and the number of confirmed deaths is around 400,000 [5].

The clinical presentation for patients with COVID-19 is nonspecific and can range from being asymptomatic to being severely ill. The most common symptoms include; fever, fatigue, dry cough, myalgia and dyspnea [1,2,6,7]. The less common symptoms include diarrhea, hemoptysis and headaches [6–8]. Due to significant limitations, including the absence of a definitive clinical diagnosis, approved vaccine, established therapy and the lack of wide scale testing, identifying and isolating infected individuals is of paramount importance to limit the spread of the disease [2,6,7].

Early radiologic investigations consistently reported that the typical computed tomography (CT) findings of COVID-19 pneumonia were bilateral ground-glass opacities (GGOs) and consolidations with a peripheral and posterior lung distribution [8,9]. Furthermore, a recently published meta-analysis reviewed the CT findings of COVID-19 patients from 34 retrospective studies. The study showed that the most common CT findings were bilateral lung opacities (73.8%; 95% CI: 65.9–81.1%) and multilobular lung involvement (67.3%; 95% CI: 54.8–78.7%) [10]. These CT findings happen to be very similar to the findings of other viruses like SARS-COV, MERS-COV, H7N9 pneumonia, H1N1 and avian influenza A (H5N1) [11,12]. Additionally, there were GGO lesions on CT without any consolidations in 45–67% of Chinese COVID-19 patients, 14–40% of MERS patients and 50% of SARS patients [12]. However, these CT findings combined together with predominant distribution in posterior and peripheral parts of the lungs were uncommon in other viral pneumonias, which makes COVID-19 unique on CT scans [12]. Some atypical findings such as pleural effusions, acute pulmonary embolism and centrilobular nodules were reported in a few cases [13].

Patients who presented with severe symptoms and were admitted to the ICU, were likely to have large areas of bilateral consolidation on CT scans. On the other hand, patients who presented with mild symptoms or were discovered incidentally by screening, when a family member presented with the illness, were likely to have GGO’s and small areas of consolidation [9]. Furthermore, data regarding the CT scan taken at different stages of the disease suggest that consolidation lesions were more prominent with less GGOs in patients with CT interval >4 days than in patients ≤4 days. This most likely indicates that consolidation increases over the course of the illness [12].

## Sensitivity of CT scans relative to reverse transcriptase PCR

There has been an ongoing debate about whether CT scans should be used alongside RT-PCR for the early detection of COVID-19. The reference standard procedure for confirming the diagnosis up until today is the reverse transcriptase PCR (RT-PCR) [2]. However, many studies have been showing that RT-PCR is not sensitive enough to be relied on solely. Some of these studies have shown that the sensitivity of RT-PCR can be as low as 30% due to limitations such as sample collection, sample transportation, kit performance and protocols used, while others showed that it can be significantly higher, up to 79% [1,2,6,14]. These RT-PCR sensitivities are based on positive results from the first test, as patients with a negative RT-PCR result were retested after 1–3 days to eliminate false-negative findings [2,14]. Because of the dissimilar RT-PCR sensitivities and shortage of RT-PCR kits, CT scans were used in China as an early test in suspected individuals [15]. Numerous studies have been conducted and the majority have concluded that CT scans are significantly more sensitive than RT-PCR for detecting positive cases, reaching up to 98% in some studies [1,2,6,14].

A systematic review and a meta-analysis assessed 16 studies and reported based on the study site, the sensitivity of chest CT was greatest in Wuhan and the sensitivity values were very close to each other (97, 96 and 99%, respectively). In the regions other than Wuhan, the sensitivity varied from 61 to 98% [16]. Additionally, two studies in the meta-analysis reported specificity of 25% (95% CI: 22–30%) and 33% (95% CI: 23–44%), respectively [16]. The reason behind this low specificity is the overlap between some of the features with other viral pneumonias [16]. Furthermore, a retrospective study of 51 COVID-19 patients showed that 50 (98%) had abnormal initial CT findings indicating a viral pneumonia, while only 36 (71%) patients had positive initial RT-PCR results [14]. A study conducted on 167 COVID-19 patients illustrated that five patients with negative RT-PCR initially had typical findings of COVID-19 pneumonia on CT [17]. Moreover, the reported RT-PCR sensitivity was as low as 60–70%, therefore, the Chinese authorities used CT scanning as a screening tool to expand the number of suspected COVID-19 cases [18]. Another study of 1014 suspected COVID-19 patients was conducted in China to determine the sensitivity and specificity of CT scans relative to RT-PCR. Initially, the results showed that 601 (59%) patients had positive RT-PCR (95% CI: 56–62%). Of these 601 patients, 583 (97%) had positive CT findings (95% CI: 95–98%) [2]. On the other hand, 308 of the remaining 413 (75%) patients with negative initial RT-PCR had positive CT findings [2]. Upon repeat RT-PCR testing and further analysis, the study concluded that 60–93% of positive cases showed positive CT findings prior to showing positive RT-PCR findings. Specifically, using serial RT-PCR measurements, the median time between positive CT scan findings and positive RT-PCR was calculated to be 1 day (range 0–7 days) [2].

Although the published data may seem in favor of the diagnostic uses of CT scanning, the current consensus disapproves of standardizing CT for screening and diagnosis [19]. Instead, studies have suggested using CT for screening patients with a negative RT-PCR result who have epidemiological and clinical features of COVID-19 [10,14,19]. Other studies, such as the one published by Lin *et al.*, state that CT scanning can differentiate between the stages of the disease, so it can be used to determine disease severity and progression [20]. Even though the results seem promising, It is not conclusive that CT scans should be used as a screening and diagnostic tool as most of the studies analyzed in this meta-analysis were single-centered. In addition, there was no clear inclusion and exclusion criteria. Hence, the presence of selection bias would have a significant impact on the sensitivity [10]. Moreover, a recently published systematic review illustrated that most current studies on CT scans’ sensitivity and specificity results are limited with bias. Therefore, current data are not sufficient to base COVID-19-screening programs using CT scans [19].

## Impact of radiologists’ perception of CT scan on diagnosis

One of the challenges associated with CT scans is the ability of radiologists to distinguish COVID-19 pneumonia from other viral pneumonias, since there are no specific guidelines about using CT scans to identify COVID-19. A retrospective analysis that consisted of 424 patients (219 COVID-19 patients and 205 non-COVID-19 patients) reported the interpretation of multiple radiologists who were blinded from the RT-PCR results. Three Chinese radiologists and four American radiologists were included in this study. Each radiologist reviewed all CT scans and reported whether the CT scan represented pneumonia caused by COVID-19, another etiology, or neither. Six radiologists had specificities of either 93 or 100% and only one had a specificity of 7%, while the sensitivities ranged from 67 to 97% [21]. Furthermore, most of the radiologists had approximately similar accuracies, ranging from 79 to 90%. Only two radiologists had significantly different accuracy rates of 53 and 97% [21]. The study concluded that radiologists were able to distinguish COVID-19 pneumonia from other viral pneumonia with high specificity and moderate sensitivity [21].

## Chest x-ray use in COVID-19

Chest x-ray (CXR) has proven to be a reliable tool in the diagnosis and management of many respiratory infections. The findings of chest x-rays mirror those of CT scans, showing bilateral peripheral consolidation [22]. However, there is insufficient literature about potential use of CXR in COVID-19. Although less sensitive than CT scans, CXRs can also be used to identify COVID-19. In one study, CXR had a sensitivity of 69%, in comparison with 91% for RT-PCR [22]. However, chest x-rays are significantly more accessible and affordable around the world, whereas CT scans are often not readily available and costly. Furthermore, the availability of portable CXR systems allows for imaging to be done within isolated rooms, significantly lowering the risk of disease spread [23].

## Guidelines by Centers for Disease Control & Prevention & American College of Radiology

In the current Centers for Disease Control & Prevention report, the use of CT for screening and diagnosing COVID-19 was regarded inappropriate due to the great variability between the reported sensitivities [24]. Moreover, the American College of Radiology mentioned in its guidelines that the use of CT in diagnosis is inappropriate. Use of CT was recommended in symptomatic patients with specific indications such as increased risk of disease progression [25]. The current consensus statement is based on the Fleischner Society, where a total of 27 panel members developed 14 key questions that established the common scenarios and recommendations where chest imaging should be used [26].

Taking numerous factors into consideration, such as severity of respiratory disease, pretest probability, risk factors for disease progression and the availability of resources, the indications for imaging were established. The consensus was that it is not indicated to do imaging to patients with suspected COVID-19 and mild symptoms unless they are at risk for disease progression. However, imaging is indicated in patients with confirmed COVID-19 and severe respiratory symptoms. In a resource-limited situation, imaging is indicated for patients that satisfy the following conditions; suspected COVID-19, moderate or severe clinical features, and high pretest probability of disease [26]. Furthermore, daily chest radiographs are not indicated in a stable-intubated patient. At last, a CT is indicated in a hypoxemic and/or functionally impaired patient, after recovery from COVID-19. It is important to note that the diagnostic algorithm always begins with either RT-PCR or the point of care rapid COVID-19 test, which are both considered first-line tests for active infection [26].

## Conclusion

Although, the sensitivity of CT scans was shown to be much higher than that of RT-PCR by some papers, there is large variability between the reported sensitivities of CT scans. Hence, the data so far are neither coherent nor reliable. Consequently, the current Centers for Disease Control and Prevention and the American College of Radiology reports recommend against the use of imaging for diagnosis, and recommend nucleic acid amplification test as the reference standard for diagnosing of COVID-19. They also state that CT could be used to determine severity and progression of the disease, or in patients with an epidemiological history and negative RT-PCR. However, the financial burden and low availability of CT scanning reduce its favorability during a pandemic. Ultimately, we recommend against the use of CT scans in symptomatic patients with positive RT-PCR given that the management of the patient will remain the same. We also agree with the recommendation regarding the use of CT scans in patients with clinical features and an epidemiological indication of COVID-19, but had a negative RT-PCR.

As demonstrated previously, there are many opinions about the use of CT in the diagnosis and management of COVID-19 (Table 1). However, COVID-19 is a novel disease, with guidelines that receive daily updates and improvements to aid in its diagnosis and management. Therefore, in order to have optimal guidelines on the role of imaging in COVID-19, more data are needed from multicentered comparative effectiveness research with a large sample size.

**Table 1.** Summary table of the characteristics and key findings of significant studies mentioned in this narrative review.

Primary study	Country	Type of study	Population size	Outcomes	Ref.
**CT scan studies**					
Zhu *et al.*	China	Meta-analysis	4121	The results of the meta-analysis showed that most patients presented bilateral lung involvement (73.8%) or multilobar involvement (67.3%). The most common changes in lesion density were GGOs (68.1%). In addition, the paper recommends using CT for screening patients with a negative RT-PCR result who have epidemiological and clinical features of COVID-19.	[10]
Xu *et al.*	China, Japan	Meta-analysis	3186	Sixteen studies were included in the meta-analysis. CT sensitivity was 92% (95% CI: 86–96%), and two studies reported specificity (25% [95% CI: 22–30%] and 33% [95% CI: 23–44%], respectively). There was substantial heterogeneity according to Cochran's Q test (p < 0.01) and Higgins I^2^ heterogeneity index (96% for sensitivity). Based on study site, the sensitivity of chest CT was great in Wuhan and the sensitivity values were very close to each other (97, 96 and 99%, respectively). In the regions other than Wuhan, the sensitivity varied from 61 to 98%.	[16]
Adams *et al.*	China, Italy, Japan	Meta-analysis	1431	Six studies were included in the meta-analysis. The sensitivity of chest CT ranged from 92.9 to 97.0%, and specificity ranged from 25.0 to 71.9%, with pooled estimates of 94.6% (95% CI: 91.9–96.4%) and 46.0% (95% CI: 31.9–60.7%), respectively. The included studies were statistically homogeneous in their estimates of sensitivity (p = 0.578) and statistically heterogeneous in their estimates of specificity (p < 0.001). Chest CT appears to have a relatively high sensitivity in symptomatic patients at high risk of COVID 19, however, specificity is poor.	[19]
Kanne *et al.*	China	Retrospective study	9720	The most common presentations were fever (98%), cough (76%) and myalgia or fatigue (44%). Dyspnea has been reported in 55% of patients. CT findings were 86% GGOs and 29% consolidation	[18]
Ai *et al.*	China	Retrospective study	1014	Fifty-nine percent had positive RT-PCR results, and 88% had positive chest CT scans. The sensitivity of chest CT in suggesting COVID-19 was 97% based on positive RT-PCR results. In patients with initial negative RT-PCR results, 75% had positive chest CT findings. In patients with negative RT-PCR results but positive chest CT scans (n = 308 patients), 48% (147/308) of patients were re-considered as highly likely cases, with 33% (103/308) as probable cases by a comprehensive evaluation.	[2]
Bai *et al.*	USA & China	Retrospective study	219	The most discriminating features for COVID-19 pneumonia included a peripheral distribution (80 vs 57%; p < 0.001), GGO (91 vs 68%; p < 0.001) and vascular thickening (58 vs 22%; p < 0.001). Three Chinese radiologists had sensitivities of 72, 72 and 94% and specificities of 94, 88 and 24%. Four US radiologists had sensitivities of 93, 83, 73 and 73% and specificities of 100, 93, 93 and 100%.	[21]
Zhao *et al.*	China	Retrospective study	101	A total of 70.2% of the patients were 21–50 years old. 78.2% had fever as the onset symptom. GGO 86.1% or mixed GGO and consolidation 64.4%, vascular enlargement in the lesion 71.3%. Lesions present on CT images were more likely to have a peripheral distribution 87.1%	[7]
Xie *et al.*	China	Retrospective study	167	The RT-PCR and CT were concordant for 2019-nCoV infection in 93%. Three percent of the patients initially had negative RT-PCR but positive chest CT with pattern consistent with viral pneumonia. Four percent had a negative chest CT with a positive RT-PCR.	[17]
Song *et al.*	China	Retrospective study	51	Chest CT showed pure GGOs in 77% of patients, GGOs with interstitial and/or interlobular septal thickening in 75% of patients, and GGOs with consolidation in 59% of patients. At chest CT, GGOs were bilateral in 88% of patients, involving the posterior lungs in 82% and the peripheral lungs in 85% of patients.	[12]
Fang *et al.*	China	Retrospective study	51	Seventy-one percent of the patients had initial positive RT-PCR for COVID-19.Twenty-four percent of the patients had COVID-19 confirmed by two RT-PCR nucleic acid tests (1–2 days), (72%) had typical CT manifestations such as GGO's and consolidation.	[14]
**Chest x-ray studies**					
Wang *et al.*	Canada	Dataset	13870	COVID-Net is one of the first open source network designs for COVID-19 detection from CXR images at the time of initial release. The introduction of COVIDx, an open access benchmark dataset. The dataset has the largest number of publicly available COVID-19 positive cases to the best of the authors’ knowledge.	[22]
Wong *et al.*	Hong Kong, China	Retrospective study	64	In a cohort of patients with COVID-19 infection and imaging follow-up, baseline chest x-ray had a sensitivity of 69%, compared with 91% for initial RT-PCR. Chest x-ray abnormalities preceded positive RT-PCR in 6/64 (9%) patients. Common chest x-ray findings mirror those previously described for CT: bilateral, peripheral, consolidation and/or GGO.	[23]

Using PubMed and Google Scholar, studies that were included were based on the following search strategy; COVID-19, CT scan, CXRs, RT-PCR and imaging. We excluded studies that did not address the topic of interest.

COVID-19: Novel coronavirus disease 2019; CT: Computed tomography; CXR: Chest x-ray; GGO: Ground-glass opacity; RT-PCR: Reverse transcriptase PCR.

## Future perspective

We hope that in 5–10 years from today, the diagnostic criteria will have changed drastically. Currently, the two potential accompanying tests to RT-PCR are CXRs and CT scans. We hope that the cost of a CT scan and the financial burden on both the patient and the government is reduced. Furthermore, higher sensitivity x-rays, new biological and radiographic technologies will hopefully be developed to aid in the diagnosis and management of the disease. At last, due to the current pandemic, the global awareness of the potential rapid spread of a highly contagious disease will have been established. Hence, a faster response will be ready and more resources will be spent on healthcare services due to the utmost importance of human lives.

**Executive summary**

**Novel coronavirus disease 2019 presentation**

- Most common presentations are fever, fatigue, dry cough, myalgia and dyspnea which are nonspecific.
- Radiological findings on computed tomography (CT): ground-glass opacities and consolidation with a peripheral and posterior lung distribution.

**Sensitivity of CT scans**

- Many studies have reported high sensitivity of CT scans and suggested its valuable use in aiding the diagnosis of novel coronavirus disease 2019 (COVID-19) alongside reverse transcriptase PCR.
- The data had huge variability and were neither accurate nor coherent.

**Impact of radiologist perception of CT scan on diagnosis**

- Most radiologists had similar sensitivities and accuracies.
- Radiologists were able to distinguish COVID-19 pneumonia from other viral pneumonia with high specificity and moderate sensitivity.

**Chest x-ray use in COVID-19**

- Chest x-rays (CXR) are more accessible and affordable worldwide.
- CXRs findings were parallel to CT scan findings, but had significantly lower sensitivity compared with CT.

**Guidelines by Centers for Disease Control & Prevention & American College of Radiology**

- Centers for Disease Control & Prevention and the American College of Radiology recommend against the use of CT as well as CXR for diagnosis and that reverse transcriptase PCR is the reference test for diagnosing of COVID-19.
